# Seipin knockout in mice impairs stem cell proliferation and progenitor cell differentiation in the adult hippocampal dentate gyrus via reduced levels of PPARγ

**DOI:** 10.1242/dmm.021550

**Published:** 2015-12-01

**Authors:** Guoxi Li, Libin Zhou, Ying Zhu, Conghui Wang, Sha Sha, Xunde Xian, Yong Ji, George Liu, Ling Chen

**Affiliations:** 1 State Key Laboratory of Reproductive Medicine, Nanjing Medical University, Nanjing 210029, China; 2 Department of Physiology, Nanjing Medical University, Nanjing 210029, China; 3 Department of Molecular Genetics, UT Southwestern Medical Center, Dallas, TX 75390, USA; 4 Key Laboratory of Cardiovascular Disease and Molecular Intervention, Atherosclerosis Research Centre, Nanjing Medical University, Nanjing 210029, China; 5 Institute of Cardiovascular Sciences, Peking University, Beijing 100191, China

**Keywords:** Seipin, *BSCL2*, Neurogenesis, Peroxisome proliferator-activated receptor gamma (PPARγ), Cell proliferation, Differentiation of progenitor cells

## Abstract

The seipin gene (*BSCL2*) was originally identified in humans as a loss-of-function gene associated with congenital generalized lipodystrophy type 2 (CGL2). Neuronal seipin-knockout (seipin-nKO) mice display a depression-like phenotype with a reduced level of hippocampal peroxisome proliferator-activated receptor gamma (PPARγ). The present study investigated the influence of seipin deficiency on adult neurogenesis in the hippocampal dentate gyrus (DG) and the underlying mechanisms of the effects. We show that the proliferative capability of stem cells in seipin-nKO mice was substantially reduced compared to in wild-type (WT) mice, and that this could be rescued by the PPARγ agonist rosiglitazone (rosi). In seipin-nKO mice, neuronal differentiation of progenitor cells was inhibited, with the enhancement of astrogliogenesis; both of these effects were recovered by rosi treatment during early stages of progenitor cell differentiation. In addition, rosi treatment could correct the decline in hippocampal ERK2 phosphorylation and cyclin A mRNA level in seipin-nKO mice. The MEK inhibitor U0126 abolished the rosi-rescued cell proliferation and cyclin A expression in seipin-nKO mice. In seipin-nKO mice, the hippocampal Wnt3 protein level was less than that in WT mice, and there was a reduction of neurogenin 1 (*Neurog1*) and neurogenic differentiation 1 (*NeuroD1*) mRNA, levels of which were corrected by rosi treatment. STAT3 phosphorylation (Tyr705) was enhanced in seipin-nKO mice, and was further elevated by rosi treatment. Finally, rosi treatment for 10 days could alleviate the depression-like phenotype in seipin-nKO mice, and this alleviation was blocked by the MEK inhibitor U0126. The results indicate that, by reducing PPARγ, seipin deficiency impairs proliferation and differentiation of neural stem and progenitor cells, respectively, in the adult DG, which might be responsible for the production of the depression-like phenotype in seipin-nKO mice.

## Abstract (precis)

**Summary:** By reducing PPARγ, neuronal seipin deficiency impairs adult neurogenesis in the hippocampal dentate gyrus to produce a depression-like phenotype in mice.

## INTRODUCTION

Congenital generalized lipodystrophy (CGL) is an autosomal recessive disorder characterized by a near-total loss of adipose tissue, severe insulin resistance, hypertriglyceridemia and fatty liver (Agarwal and Garg, 2003). Genome-wide linkage analysis has identified two loci for CGL, each of which causes a different type of the disease: CGL1 results from mutation in the 1-acylglycerol-3-phosphate-O-acyl transferase 2 (*AGPAT2*) gene and CGL2 from mutation in the Berardinelli-Seip congenital lipodystrophy 2 (*BSCL2*) gene, which encodes seipin (Magre et al., 2001). Individuals with CGL2 show more severe lipoatrophy compared with those with CGL1, but the prevalence of metabolic abnormalities is similar in the two subtypes (Simha and Garg, 2003). An interesting phenotypic difference between individuals with CGL1 and CGL2 is that those with CGL2 have an increased prevalence of mild mental retardation, which is not usually observed in CGL1 (Agarwal et al., 2003). Clinical studies (Rajab et al., 2003; Van Maldergem et al., 2002) have reported the delayed cognitive development and intellectual impairment in individuals with CGL2. The seipin gene was originally identified as a loss-of-function mutation in individuals with CGL2 (Van Maldergem et al., 2002). Seipin is highly expressed in the brain cortex, cerebellum, hippocampus and hypothalamus (Garfield et al., 2012; Magre et al., 2001). Ebihara et al. (2015) have recently reported that seipin-knockout rats show impaired spatial working memory. In addition, we have observed the anxiety- and depression-like phenotypes in systemic seipin-knockout (seipin-sKO) and neuron-specific seipin-knockout (seipin-nKO) mice, but not in fat-specific seipin-knockout mice (Zhou et al., 2014). Therefore, it has been speculated that seipin is necessary for developmental processes in the brain.

Seipin, as an exclusively endoplasmic reticulum (ER)-resident N-glycosylated protein, plays a role in the generation of peroxisome proliferator-activated receptor gamma (PPARγ) (Fei et al., 2011). PPARγ is highly expressed in the brain of embryos (Wada et al., 2006) and adult animals (Denner et al., 2012) such as mice or rats. Several lines of evidence suggest that seipin depletion can reduce the expression of PPARγ in mesenchymal stem cells (Chen et al., 2009), murine embryonic fibroblasts and stromal vascular cells (Chen et al., 2012), and mouse white adipose tissue (Liu et al., 2014). Recently, the reduction of PPARγ has been found in embryonic fibroblasts of seipin-deficient mice (Prieur et al., 2013) and in the hippocampus of seipin-nKO mice (Zhou et al., 2014). Growing evidence suggests that the downregulation of PPARγ is tightly associated with a high risk for depression development (Yaffe et al., 2008). The PPARγ agonist rosiglitazone (rosi) can effectively improve the depression-like behaviors in seipin-nKO mice (Zhou et al., 2014).

**TRANSLATIONAL IMPACT**

**Clinical issue**

Congenital generalized lipodystrophy (CGL) is an autosomal recessive disorder characterized by loss of adipose tissue and other metabolic abnormalities. Individuals with CLG type 2 (CGL2), which is associated with a loss-of-function mutation in seipin (a regulator of lipid catabolism), also often show delayed cognitive development and develop affective disorders such as depression. The mechanisms underlying mental retardation and mood changes in these individuals are poorly understood. In a recent study, neuronal seipin-knockout (seipin-nKO) mice displayed a depression-like phenotype and had reduced hippocampal levels of peroxisome proliferator-activated receptor gamma (PPARγ), a regulator of neuronal proliferation and differentiation. Hippocampal neurogenesis is known to be important for mood control and antidepressant efficacy, and seipin is expressed in neural stem cells and progenitor cells in the subgranular zone of adult hippocampal dentate gyrus. Thus, seipin deficiency might cause mental retardation in individuals with CGL2 by reducing PPARγ levels and thereby affecting adult neurogenesis.

**Results**

Here, the authors use the seipin-nKO mouse to investigate the influence of seipin deficiency on the process of adult neurogenesis. They show that the proliferative capability of stem cells and the neuronal differentiation of progenitor cells are significantly reduced in seipin-nKO mice, but that these phenotypes can be rescued by treatment with the PPARγ agonist rosiglitazone. Seipin deficiency, they show, causes inactivation of hippocampal ERK2 signaling and reduced cyclin A expression, thereby impairing the proliferative capability of stem cells. In addition, seipin deficiency reduces hippocampal Wnt3 levels, which leads to downregulation of Neurog1 and NeuroD1 and depression of the neuronal differentiation of progenitor cells. Notably, the authors show that treatment with rosiglitazone alleviates the depression-like phenotype of seipin-nKO mice and that this effect of rosiglitazone is blocked by the inhibition of ERK signaling.

**Implications and future directions**

These results suggest that the reduction of PPARγ levels caused by seipin deficiency impairs neurogenesis in the hippocampal dentate gyrus in mice and might be responsible for the production of the depression-like phenotype in seipin-nKO mice. Thus, the intellectual deficiency seen in individuals with CGL2 might, at least partly, be the result of impairment of adult neurogenesis in the hippocampal dentate gyrus. Although further work is needed to confirm and extend these findings, the present study raises the possibility that the therapeutic use of PPARγ agonists might help to limit or reverse the intellectual deficiency seen in individuals with CGL2 by reinstating hippocampal neurogenesis.

A large body of evidence has established that the mammalian brain produces continuously newborn neurons in the hippocampal dentate gyrus (DG) throughout adult life (Toni et al., 2008). These newly generated neurons can migrate into the granular cell layer of the DG and integrate into hippocampal synaptic circuits. It is widely accepted that hippocampal neurogenesis is required for mood control (Petrik et al., 2012) and cognitive performance (Rola et al., 2004). Clinical trials have proved that the hippocampal volume in individuals with depression is smaller than normal age-matched subjects (Videbech and Ravnkilde, 2004). Selective impairment of adult neurogenesis by telomerase inhibitor could cause the depression-like behaviors in mice (Zhou et al., 2011). Irradiation of young animals impairs hippocampal neurogenesis and is associated with cognitive deficits (Rola et al., 2004). Importantly, we have observed high expression of seipin in neural stem and progenitor cells in the hippocampal subgranular zone (SGZ) of adult mice (Zhou et al., 2014; and in this study). PPARγ has also been identified in stem cells (Wada et al., 2006). The inhibition or deficiency of PPARγ affects the proliferation and differentiation of stem cells (Wada et al., 2006). The reduction of PPARγ by seipin knockdown alters the differentiation of mouse embryonic fibroblasts (Prieur et al., 2013). The knockdown of seipin in 3T3-L1 and C3H10T1/2 cell lines suppresses the terminal adipocyte differentiation (Chen et al., 2009; Payne et al., 2008). The knockdown of PPARγ in neuronal cells significantly decreases the cell growth rate (Katayama et al., 2005). Thus, it should be interesting to explore whether the seipin deficiency, through reducing PPARγ levels, affects adult neurogenesis, leading to the mental retardation.

In the present study, we used male seipin-nKO mice and examined the influence of seipin deficiency on the process of adult neurogenesis in the hippocampal DG, including the proliferation of stem cells, differentiation of progenitor cells, neurite growth and the survival of newborn neurons. In addition, we investigated the reduced-PPARγ-related molecular mechanisms underlying the abnormal neurogenesis in seipin-nKO mice. Finally, we explored the causal link between the abnormal neurogenesis in seipin-nKO mice and their depression-like phenotypes. Our results indicate that seipin deficiency, by reducing the level of PPARγ, suppresses the proliferation of stem cells and neuronal differentiation of progenitor cells, causing depression-like phenotype.

## RESULTS

### Seipin deficiency reduces PPARγ levels, which suppresses proliferation of stem cells in the DG

**Fig. 1.**
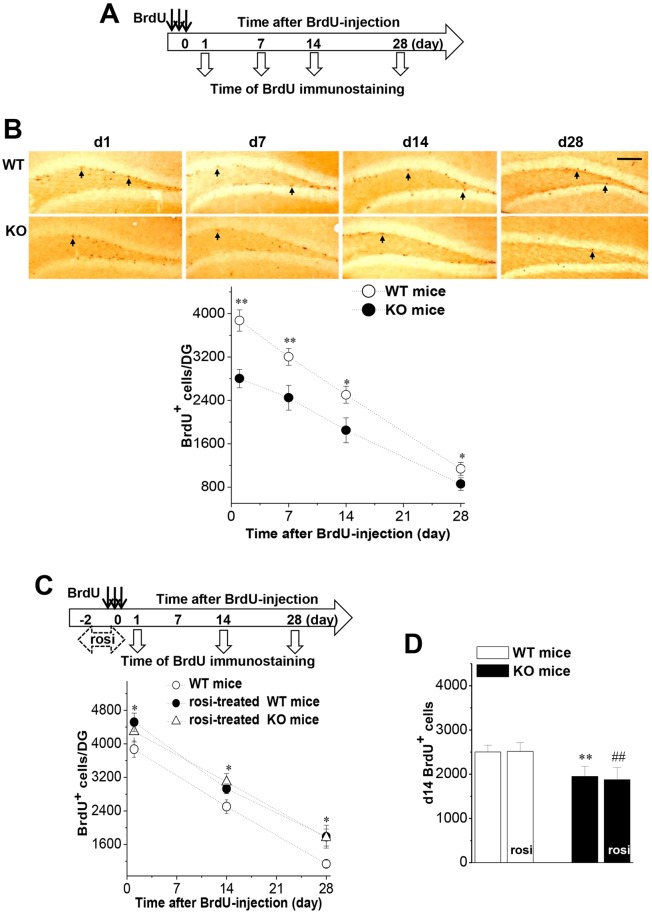
**Seipin deficiency suppresses adult neurogenesis in the DG by reducing PPARγ.** (A) Time chart of experimental procedure. Black arrows (↓) indicate the time of BrdU injection. Hollow arrows indicate the time of BrdU immunostaining. (B) Each point in the graph represents the group mean of BrdU^+^ cells at day 1 after the last BrdU injection (d1), d7, d14 and d28 in seipin-nKO mice and wild-type (WT) mice. Representative images of BrdU immunostaining in seipin-nKO mice (KO) and WT mice. Black arrows indicate BrdU^+^ cells. Scale bar: 200 μm. *P<0.05, **P<0.01 vs WT mice. (C) Influence of rosi treatment for 3 days before the last injection of BrdU on the number of d1, d14 or d28 BrdU^+^ cells in WT mice and seipin-nKO mice treated with rosi. *P<0.05 vs WT mice. (D) Effects of rosi treatment at d7-d12 after BrdU injection on the d14 BrdU^+^ cells in seipin-nKO mice and WT mice. **P<0.01 vs WT mice; ^##^*P*<0.01 vs seipin-nKO mice.

To investigate the influence of seipin deficiency on the cell proliferation and survival of newborn neurons, BrdU immunostaining was performed on mice sacrificed on day 1 (d1), d7, d14 and d28 after the last injection of BrdU (Fig. 1A). This timing roughly corresponds to the mitosis of stem cells (24 h), the survival of newborn cells (day 4-10), and mature neuronal marker expression (week 2-3) in rats and mice (Galea, 2008). In comparison to wild-type (WT) mice, the numbers of d1 (*P*<0.01, *n*=8; Fig. 1B), d7 (*P*<0.01, *n*=8), d14 (*P*<0.05, *n*=8) or d28 (*P*<0.05, *n*=8) BrdU^+^ cells were reduced approximately 25-30% in seipin-nKO mice. Because the volume of the DG had no significant difference between seipin-nKO mice and WT mice (data not shown), the total number of BrdU^+^ cells normalized to the DG volume was still less in seipin-nKO mice.

In comparison to vehicle-treated controls, treatment of WT mice with the PPARγ agonist rosi [5 mg/kg body weight, per os (p.o.)] for 3 days before the last injection of BrdU (Fig. 1C) caused an approximate 15% increase in the number of both d1 BrdU^+^ cells (*P*<0.05, *n*=8) and d14 and d28 BrdU^+^ cells (*P*<0.05, *n*=8). In seipin-nKO mice, the rosi treatment led to an approximate 55% increase in the number of d1 BrdU^+^ cells (*P*<0.01, *n*=8), with an equal increase in the number of d14 and d28 BrdU^+^ cells (*P*<0.01, *n*=8), which abolished their difference from WT mice (*P*>0.05, *n*=8). However, when administered on d7-d12 after BrdU injection, rosi had no effect on the number of d14 BrdU^+^ cells in seipin-nKO mice (*P*>0.05, *n*=8; Fig. 1D) or WT mice (*P*>0.05, *n*=8).

### Seipin deficiency suppresses neuronal differentiation of progenitor cells via reduced PPARγ

**Fig. 2.**
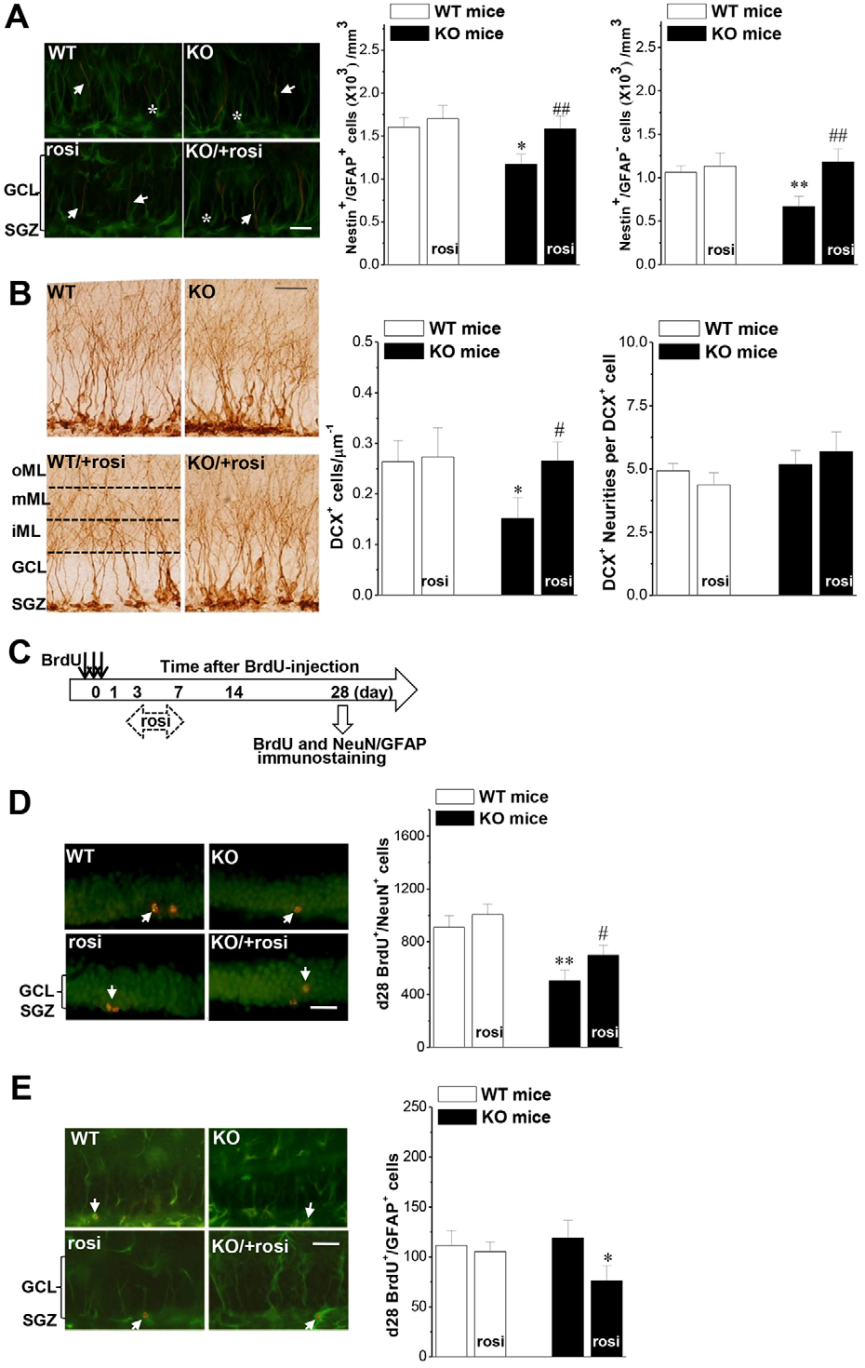
**By reducing PPARγ, seipin deficiency suppresses neuronal differentiation of progenitor cells in the DG.** (A) Influence of seipin deficiency on the differentiation of neuronal stem cells. Bar graphs show the density of nestin^+^/GFAP^+^ cells or nestin^+^/GFAP^−^ cells in seipin-nKO mice and WT mice treated with rosi or vehicle for 3 days before the last injection of BrdU. Representative images of nestin/GFAP immunostaining are shown. GCL indicates the granule cell layer. SGZ indicates the subgranular zone. White arrows indicate nestin^+^/GFAP^+^ cells; white asterisks indicate nestin^+^/GFAP^−^ cells. Scale bar: 25 μm. *P<0.05 vs WT mice; **P<0.01 vs WT mice; ^##^*P*<0.01 vs seipin-nKO mice. (B) Bar graph shows mean number of DCX^+^ cells and density of DCX^+^ fibers in seipin-nKO mice and WT mice treated with rosi or vehicle for 3 days before the last injection of BrdU. Representative images of DCX immunostaining are shown. The molecular layer (ML) of the DG was divided into three subregions: the inner (iML), middle (mML) and outer (oML) ML. Scale bar: 50 μm. *P<0.05 vs WT mice; ^#^*P*<0.05 vs seipin-nKO mice. (C) Time chart of experimental procedure. (D,E) Influence of seipin deficiency on neuronal differentiation of progenitor cells. Bar graph shows mean number of d28 (28 days after last BrdU injection) BrdU^+^/NeuN^+^ cells and d28 BrdU^+^/GFAP^+^ cells in seipin-nKO mice and WT mice. Rosi was administered on d3-d7 after the last injection of BrdU. Representative images of BrdU/NeuN and BrdU/GFAP immunostainings are shown. White arrows indicate BrdU^+^/NeuN^+^ cells and BrdU^+^/GFAP^+^ cells. Scale bars: 50 μm. (D) **P<0.01 vs WT mice; ^#^*P*<0.05 vs seipin-nKO mice; (E) *P<0.05 vs seipin-nKO mice.

The adult DG contains at least two types of proliferating immature cells that can be labeled by BrdU: type 1 is nestin^+^/GFAP^+^ radial glia-like cells, which are stem cells; type 2 is nestin^+^/GFAP^−^ cells, which are more likely to be progenitor cells and can differentiate into type 3 (DCX expression) neuroblasts (Zhao et al., 2008). In comparison to WT mice, the number of nestin^+^/GFAP^+^ cells (*P*<0.05, *n*=8; Fig. 2A) or nestin^+^/GFAP^−^ cells (*P*<0.01, *n*=8) in seipin-nKO mice was reduced, which was recovered by rosi treatment for 3 days before the last injection of BrdU (*P*<0.05, *n*=8). Additionally, a similar reduction in the number of DCX-positive (DCX^+^) cells was found in seipin-nKO mice (*P*<0.05, *n*=8; Fig. 2B) ,without the changes in the number of DCX^+^ fibers per DCX^+^ cell (density of DCX^+^ fibers) (*P*>0.05, *n*=8). Rosi treatment for 3 days before the last injection of BrdU in seipin-nKO mice increased the number of DCX^+^ cells (*P*<0.05, *n*=8).

Sequentially, we further estimated the relative proportions of d28 BrdU^+^ cells expressing NeuN or GFAP. The number of d28 BrdU^+^/NeuN^+^ cells was reduced in seipin-nKO mice compared to WT mice (*P*<0.01, *n*=8; Fig. 2D), whereas the number of BrdU^+^/GFAP^+^ cells revealed no differences between both groups (*P*>0.05, *n*=8; Fig. 2E). The percentage of BrdU^+^/NeuN^+^ cells against total BrdU^+^ cells in seipin-nKO mice (58.7±4.61%) was less than that in WT mice (79.8±4.15%, *P*<0.05, *n*=8), whereas the percentage of BrdU^+^/GFAP^+^ cells in seipin-nKO mice (14.6±4.61%) was higher than control (9.7±4.32%, *P*<0.05, *n*=8). Compared with untreated seipin-nKO mice, rosi treatment on d3-d7 after the last injection of BrdU (Fig. 2C), an early stage of neuronal differentiation, increased the number of BrdU^+^/NeuN^+^ cells (*P*<0.05, *n*=8; Fig. 2D) and reduced the number of BrdU^+^/GFAP^+^ cells (*P*<0.05, *n*=8; Fig. 2E) at d28 in seipin-nKO mice, recovering the proportion of BrdU^+^/NeuN^+^ cells (81.4±3.17%) and BrdU^+^/GFAP^+^ cells (9.5±2.03%) in seipin-nKO mice to WT levels (vs WT mice *P*>0.05, *n*=8).

### Seipin deficiency suppresses ERK activation via reduced PPARγ

**Fig. 3.**
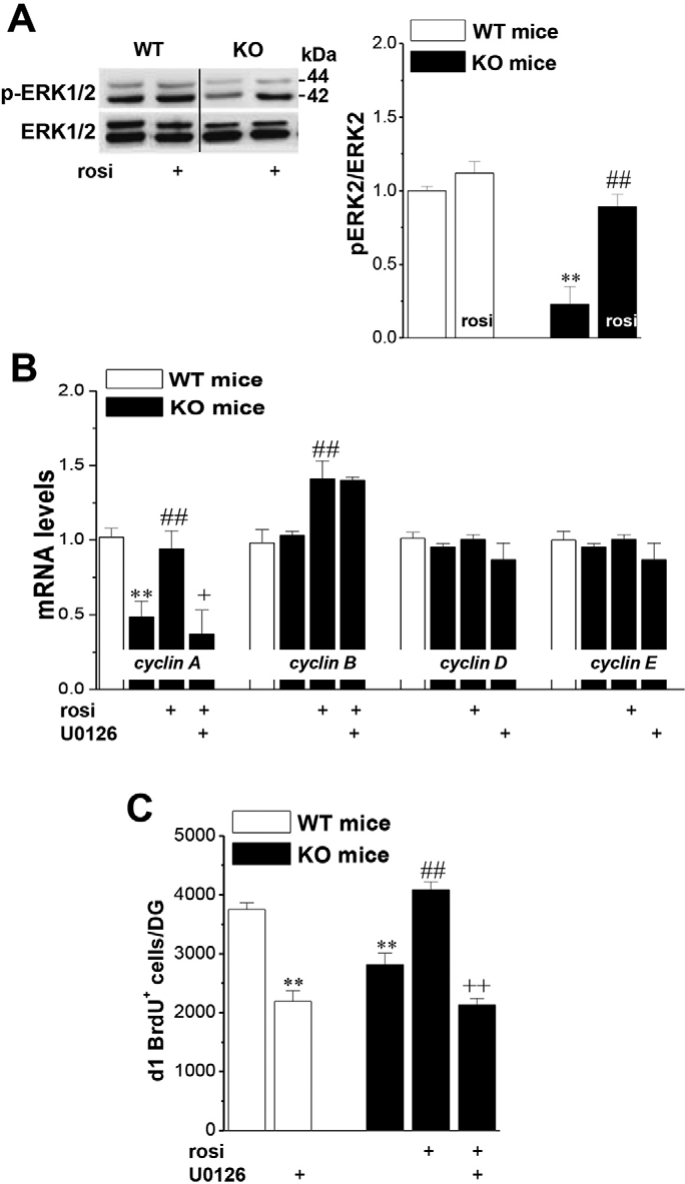
**Seipin deficiency suppresses phospho-ERK2 and cyclin A expression.** (A) Densitometric values of phospho-ERK2. **P<0.01 vs WT mice; ^##^*P*<0.01 vs seipin-nKO mice. (B) Levels of cyclin A, cyclin B, cyclin D and cyclin E mRNA normalized by control values. **P<0.01 vs WT mice; ^##^*P*<0.01 vs seipin-nKO mice; ^+^*P*<0.05 vs rosi-treated seipin-nKO mice. (C) Effect of the MEK inhibitor U0126 on rosi-protected neurogenesis. Bar graph shows mean number of d1 (1 day after last BrdU injection) BrdU^+^ cells in seipin-nKO mice and WT mice treated with U0126, rosi or rosi+U0126. **P<0.01 vs WT mice; ^##^*P*<0.01 vs seipin-nKO mice; ^++^*P*<0.01 vs rosi-treated seipin-nKO mice.

The inhibition of PPARγ has been reported to block ERK2 activation (Denner et al., 2012). To explore the mechanisms underlying the seipin-deficiency-suppressed cell proliferation, we examined hippocampal ERK1/2 phosphorylation (phospho-ERK1/2) and the expression of cyclin family members that are known to be important for the regulation of cell proliferation. Western blot analysis revealed that the level of phospho-ERK1/2 was significantly reduced in seipin-nKO mice compared to WT mice (*P*<0.01, *n*=8; Fig. 3A), and the level was normalized by the rosi treatment for 3 days before the last injection of BrdU (*P*<0.01, *n*=8).

Similarly, the level of cyclin A mRNA was markedly reduced in seipin-nKO mice compared with WT (*P*<0.01, *n*=8; Fig. 3B) without affecting other cyclin family members such as the levels of cyclin B, cyclin D and cyclin E mRNA (*P*>0.05, *n*=8). Rosi treatment for 3 days before the last injection of BrdU corrected the downregulation of cyclin A expression (*P*<0.01, *n*=8), which was sensitive to the infusion [intracerebroventricular injection (i.c.v.)] of the MEK inhibitor U0126 (*P*<0.05, *n*=8), and increase the level of cyclin B mRNA (*P*<0.01, *n*=8) in seipin-nKO mice. Similarly, U0126 inhibited the increase in cyclin A mRNA induced by rosi in seipin-nKO mice (*P*<0.05, *n*=8). In addition, the infusion of U0126 not only blocked the increase in d1 BrdU^+^ cells induced by rosi in seipin-nKO mice (*P*<0.01, *n*=8; Fig. 3C) but also reduced the number of d1 BrdU^+^ cells in WT mice (*P*<0.05, *n*=8).

### Seipin deficiency suppresses Wnt3 signaling via reduced PPARγ

**Fig. 4.**
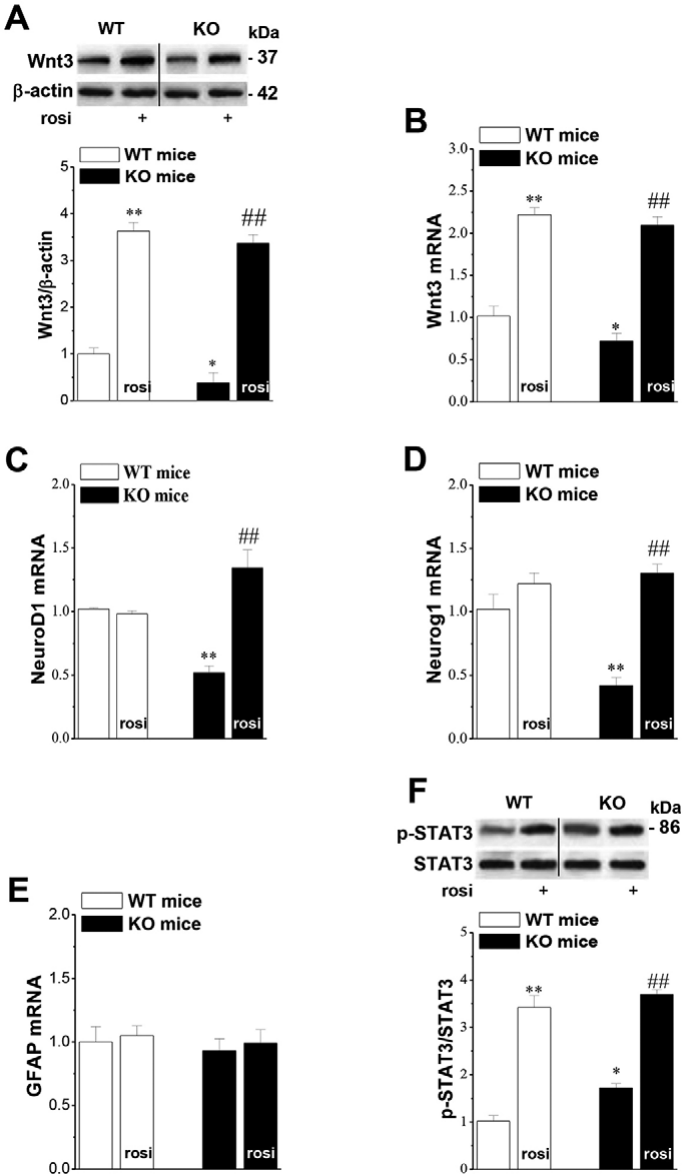
**Influence of seipin deficiency on the Wnt3 signaling pathway.** (A) Densitometric values of Wnt3. *P<0.05, **P<0.01 vs WT mice; ^##^*P*<0.01 vs seipin-nKO mice. (B) Bar graph shows mean level of *Wnt3* mRNA. *P<0.05, **P<0.01 vs WT mice; ^##^*P*<0.01 vs seipin-nKO mice. (C-E) Bar graphs show the levels of *NeuroD1* mRNA, *Neurog1* mRNA and *GFAP* mRNA normalized by control values in WT mice and seipin-nKO mice. (C) **P<0.01 vs WT mice; ^##^*P*<0.01 vs seipin-nKO mice; (D) **P<0.01 vs WT mice; ^##^*P*<0.01 vs seipin-nKO mice. (F) Densitometric values of phospho-STAT3 (Tyr705). *P<0.05 vs WT mice, **P<0.01 vs WT mice; ^##^*P*<0.01 vs seipin-nKO mice.

The activation of PPARγ plays a key role in modulating cell differentiation, which depends on the Wnt3 signaling pathway (Ross et al., 2000). To investigate the molecular mechanisms of the impaired differentiation of progenitor cells by reduced PPARγ, we examined hippocampal Wnt3 expression in seipin-nKO mice. The levels of Wnt3 protein (*P*<0.05, *n*=8; Fig. 4A) or *Wnt3* mRNA (*P*<0.05, *n*=8; Fig. 4B) were reduced in seipin-nKO mice compared to WT mice, and this reduction was recovered by rosi treatment for 3 days before the last injection of BrdU (*P*<0.01, *n*=8). Rosi treatment also elevated the levels of Wnt3 protein (*P*<0.01, *n*=8) and *Wnt3* mRNA (*P*<0.01, *n*=8) in WT mice.

NeuroD1 and Neurog1 are downstream effectors of Wnt3 in the process of neurogenesis (Hsieh, 2004). In comparison with WT mice, seipin-nKO mice showed a significant reduction in the levels of *NeuroD1* (*P*<0.01, *n*=8; Fig. 4C) and *Neurog1* (*P*<0.01, *n*=8; Fig. 4D) mRNA, without change in the level of *GFAP* mRNA (*P*>0.05, *n*=8; Fig. 4E). Rosi treatment for 3 days before the last injection of BrdU rescued the reduction of *NeuroD1* (*P*<0.01, *n*=8) and *Neurog1* (*P*<0.01, *n*=8) mRNA in seipin-nKO mice, but it had no effect in WT mice (*P*>0.05, *n*=8).

PPARγ has been reported to specifically enhance phospho-STAT3 (Kinouchi et al., 2012). Notably, in the present study, the hippocampal phospho-STAT3 level was higher in seipin-nKO mice than in WT mice (*P*<0.01, *n*=8; Fig. 4F) without changing STAT3 protein level (*P*>0.05, *n*=8). Rosi treatment for 3 days before the last injection of BrdU further elevated the level of phospho-STAT3 in seipin-nKO mice (*P*<0.01, *n*=8) and enhanced the phospho-STAT3 in WT mice (*P*<0.01, *n*=8).

### Neurogenesis decrease in seipin-nKO mice leads to depression

**Fig. 5.**
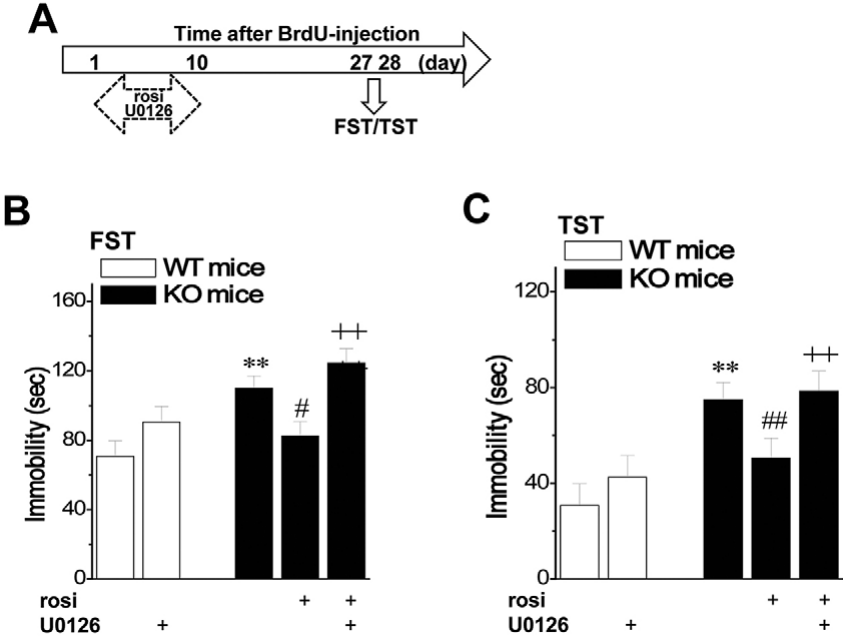
**Relationship between the decrease in neurogenesis and the depressive-like phenotype in seipin-nKO mice.** (A) Time chart of the experimental procedure. Horizontal hollow arrows indicate the time of U0126 administration. (B,C) Influence of U0126 on rosi-improved depression-like behaviors in seipin-nKO mice. Bars represent immobile time during the FST and TST in seipin-nKO mice and WT mice. (B) **P<0.01 vs WT mice; ^#^*P*<0.05 vs seipin-nKO mice; ^++^*P*<0.01 vs rosi-treated seipin-nKO mice; (C) **P<0.01 vs WT mice; ^##^*P*<0.01 vs seipin-nKO mice; ^++^*P*<0.01 vs rosi-treated seipin-nKO mice.

Rosi treatment for 10 days after the last BrdU injection in seipin-nKO mice (Fig. 5A) prevented the prolonged immobility in the forced swim test (FST; *P*<0.05, *n*=12; Fig. 5B) and tail suspension test (TST; *P*<0.01, *n*=12; Fig. 5C). To test whether the decrease in neurogenesis in seipin-nKO mice is crucial for the production of the depressive-like phenotype, seipin-nKO mice were treated via injection (i.c.v.) with U0126, which selectively blocked the rosi-protected neurogenesis. As expected, the co-administration of U0126 abolished the antidepressant effects of rosi in seipin-nKO mice (*P*<0.01, *n*=12). The treatment of WT mice with U0126 slightly increased the immobility time in the FST and TST, but the group comparison failed to reach significance (*P*>0.05, *n*=12).

## DISCUSSION

Seipin is abundantly expressed in the stem and progenitor cells of the hippocampal DG (Fig. S1A). The present study provides, for the first time, *in vivo* evidence that seipin deficiency, through reduced PPARγ, impairs the proliferation of stem cells and differentiation of progenitor cells in the hippocampal DG, and this impairment might be responsible for the production of the depression-like phenotype in seipin-nKO mice.

**Fig. 6.**
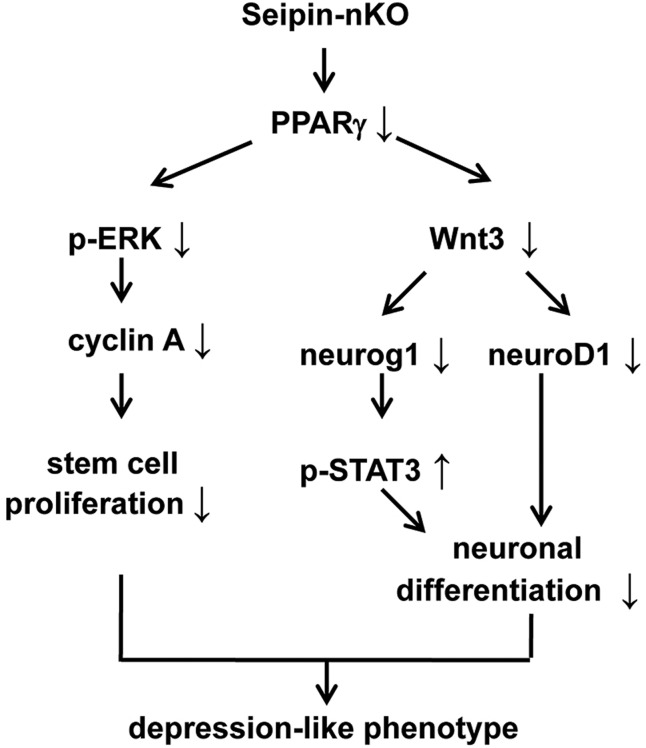
**The hypothesis of molecular mechanisms underlying the seipin-deficiency-induced impairment of adult neurogenesis in the hippocampal DG. ↑**, increase; **↓**, decrease.

Interestingly, hippocampal PPARγ expression is reduced in male seipin-nKO mice, but not in female seipin-nKO mice, probably owing to the enhancing effect of estrogen on PPARγ expression (Zhou et al., 2014). Rosi treatment can recover the reduction of PPARγ expression through increasing the *PPARγ* gene transcripts in Tg2576 mice (Denner et al., 2012). Male seipin-nKO mice showed a significant decrease in the numbers of d1 BrdU^+^ cells and nestin^+^ cells, which was rescued by rosi treatment. By contrast, the proliferative capability of stem cells in female seipin-nKO mice failed to be affected (Fig. S2). Neuronal PPARγ-knockout leads to increased ischemic brain damage, which has no sexual difference (Zhao et al., 2009). The absence of PPARγ has been reported to inhibit the self-renewal capability of stem cells (Wada et al., 2006). The inhibition of PPARγ downregulates ERK2 activation (Denner et al., 2012). The activation of PPARγ can stimulate the cell cycle via upregulation of cyclin family members (Yam et al., 2002). PPARγ-induced ERK activation can accelerate the cell cycle via increasing cyclin B level (Cimini and Ceru, 2008). In seipin-nKO mice, the phospho-ERK and expression of cyclin A, but not cyclin B, were remarkably reduced. Although the rosi treatment in seipin-nKO mice could increase the phospho-ERK and the levels of cyclin A and cyclin B mRNA, only the rosi-increased cyclin A was sensitive to the MEK inhibitor U0126. Moreover, U0126 could block rosi-recovered proliferative capability of stem cells in seipin-nKO mice. Thus, it is conceivable that the reduced PPARγ in seipin-nKO mice suppresses the cell proliferation through inactivation of ERK to reduce the expression of cyclin A (Fig. 6).

Another principal observation in this study is that the seipin deficiency, through reduced PPARγ, suppresses the neuronal differentiation of progenitor cells in the DG. This conclusion is deduced mainly from the following observations: the amounts of nestin^+^/GFAP^−^ cells and DCX^+^ cells were significantly reduced in seipin-nKO mice, which was rescued by the rosi treatment. The numbers of d28 BrdU^+^ and BrdU^+^/NeuN^+^ cells were reduced in seipin-nKO mice, but the number of BrdU^+^/GFAP^+^ cells had no difference from WT mice. The relative proportion of BrdU^+^/NeuN^+^ cells was lower, whereas the proportion of BrdU^+^/GFAP^+^ cells was higher, in seipin-nKO mice than in WT mice. The rosi treatment during the early stage of neuronal differentiation increased the number of BrdU^+^/NeuN^+^ cells and corrected the normal proportions of BrdU^+^/NeuN^+^ cells and BrdU^+^/GFAP^+^ cells in seipin-nKO mice, although it did not increase the absolute number of d28 BrdU^+^ cells. PPARγ can enhance Wnt3 expression (Fuentealba et al., 2004; Inestrosa et al., 2005) in stem or progenitor cells in the adult DG (Zhou et al., 2004). In the course of neurogenesis, the increasing Wnt3A can induce the expression of NeuroD1 (Kuwabara et al., 2009). NeuroD1 is selectively expressed in dividing neural progenitors and in immature granule neurons in the adult DG (Hsieh, 2004). The inhibition of Wnt signaling or the deletion of NeuroD1 causes the deficits in the hippocampal neurogenesis (Gao et al., 2009). The downregulation of Wnt3 and NeuroD1 was observed in seipin-nKO mice, which was recovered by the rosi treatment. On the other hand, the downregulation of Wnt3 signaling reduces the expression of Neurog1 (Luo et al., 2010). Neurog1 is an early initiator of neuronal differentiation and an inhibitor of glial differentiation, and its downregulation can reduce neuronal differentiation and increase glial differentiation (Liu et al., 2010; Luo et al., 2010) by inhibiting JAK/STAT signaling (Sun et al., 2001). Indeed, seipin-nKO mice showed the reduction of Neurog1 and the elevation of phospho-STAT3. Wei et al. (2014) reported a transient increase of phospho-STAT3 during the early stages of neuronal differentiation. The deletion of STAT3 can promote neurogenesis and inhibit astrogliogenesis through downregulation of notch-hes signaling (Gao et al., 2009; Gu et al., 2005). Thus, it is proposed that the downregulation of Neurog1 in seipin-nKO mice can enhance phospho-STAT3 to suppress the neuronal differentiation of progenitor cells (Fig. 6). There were conflicting results showing that the rosi treatment in seipin-nKO mice further elevated the phospho-STAT3 level, although it recovered the Wnt3 and Neurog1 levels. In addition, the rosi-increased phospho-STAT3 level in WT mice did not alter the differentiation of progenitor cells. Therefore, another possibility is that the rosi-recovered neuronal differentiation of progenitor cells in seipin-nKO mice might arise from increasing the levels of NeuroD1 and Neurog1, rather than inhibiting phospho-STAT3.

The treatment with PPARγ antagonists in neuronal stem cells can cause nuclear condensation and the increase of cleaved caspase-3 (Wada et al., 2006). Although the number of d28 BrdU^+^ cells was less in seipin-nKO mice than in WT mice, the percentage of BrdU^+^ cells from 1 day to 4 weeks after birth in seipin-nKO mice, which generally declined over this time (100% d1→87.4% d7→65.9% d14→30.5% d28), did not differ significantly from WT mice (82.7% d7→64.6% d14→29.3% d28). After rosi rescued cell proliferation in seipin-nKO mice, the reduction of d14 and d28 BrdU^+^ cells could be corrected. However, the rosi treatment on d7-d12 after BrdU injection had no effect on the reduction of d14 BrdU^+^ cells in seipin-nKO mice. In addition, the neuronal PPARγ deficiency alone in mice is not sufficient to cause neuronal loss in the hippocampus, although it reduces the ability of neurons to handle oxidative insult (Zhao et al., 2009). Therefore, seipin deficiency is unlikely to impair the survival of newly generated neurons in the DG. Further investigations are required to clarify the details.

The cause-result relationship between hippocampal neurogenesis and cognitive performance or mood control is supported by anatomical and functional observations. The newly generated neurons are integrated into hippocampal circuitry within 3-4 weeks after birth (Zhao et al., 2006). In comparison to mature neuronal synapses, the newborn synapses have lower activation thresholds and higher resting potentials (Schmidt-Hieber et al., 2004). Thus, the adult-generated synapses are crucial for the hippocampal function and output (Ming and Song, 2011). The basolateral amygdaloid complex, which is associated with emotionality, receives synaptic inputs from the hippocampus (Supcun et al., 2012). Although there is not enough evidence to draw the conclusion, our results indicate that the impairment of neurogenesis is able to induce the depressive-like phenotype in seipin-nKO mice (Fig. 6), as suggested by the following results. The rosi treatment in seipin-nKO mice could improve the depressive-like behaviors and the neurogenesis deficits, which was abolished by the MEK inhibitor U0126. In addition, female seipin-nKO mice did not show the depression-like behaviors and neurogenesis deficits, because estrogen is able to correct the reduction of PPARγ expression (Zhou et al., 2014). The changing afferent and efferent connectivity of the hippocampus along the longitudinal axis in rodents and primates indicates discrete functions of the dorsal and ventral hippocampus in learning and emotionality (Moser and Moser, 1998). The dorsal DG receives projections arising in the lateral and caudomedial portion of the entorhinal cortex, whereas the ventral DG receives inputs from the most rostromedial region of the entorhinal cortex (Dolorfo and Amaral, 1998). Lesion in the dorsal hippocampus affects spatial learning and memory (Moser et al., 1995), whereas lesion of the ventral hippocampus leads to anxiety- and depressive-like behaviors (McHugh et al., 2004). The number of newborn neurons in either the dorsal DG or ventral DG was decreased in seipin-nKO mice. Moreover, seipin-knockout rats show spatial memory impairment (Ebihara et al., 2015), Thus, the results indicate that the hippocampal neurogenesis deficits in seipin-nKO mice can affect spatial cognitive function (Fig. 6).

In comparison to WT mice, the levels of hippocampal *PPARγ* mRNA and PPARγ protein are significantly reduced in male seipin-nKO mice (Zhou et al., 2014). The elevation of phosphatidic acid (PA) has been reported in *seipin*-knockdown yeast (Szymanski et al., 2007). Liu et al. (2014) reported the significant increase of PA in adipose-specific seipin-knockout mice. Similarly, we observed the accumulation of PA in hippocampus of male and female seipin-nKO mice (Fig. S3). The seipin knockdown impairs the differentiation of preadipocyte cells, accompanied by an early suppression of PPARγ expression (Chen et al., 2009). Because the PPARγ agonist can reverse the defective differentiation caused by seipin depletion, it is conceivable that seipin functions upstream of PPARγ. Thus, it is proposed that the seipin deficiency causes the accumulation of PA in neuronal cells to suppress the expression of PPARγ. On the other hand, seipin is predicted to span the ER membrane twice, with both the N- and C-termini in the cytoplasm and a large luminal loop (Ito et al., 2008). Missense mutations (N88S and S90L) of seipin produce the unfolded proteins response (UPR) to induce ER stress (Yagi et al., 2011). However, the protein levels of the ER-stress markers BIP and CHOP are not increased in the hippocampus of seipin-nKO mice (Zhou et al., 2014). Any apparent phenotypes of motor neuron disease that is associated with gain-of-toxic-function mutation in the N-glycosylation site of seipin (Ito and Suzuki, 2009) were not observed in seipin-nKO mice (Zhou et al., 2014), seipin-knockout rats or humans with CGL2 (Ebihara et al., 2015). Thus, the decreased PPARγ expression in seipin-nKO mice is unlikely to rise from the ER stress.

In summary, the results in this study will bring new insights into the physiological function of seipin in the process of adult hippocampal neurogenesis. Although much more work needs to be performed in the future, the anxiety- and depressive-like behaviors in seipin-nKO mice, at least partly, relies on the abnormal proliferation and differentiation of adult neural stem and progenitor cells in the hippocampal DG through reduced PPARγ. However, the affective disorder is not observed in humans with CGL2. Mood disorders are a major concern for people with intellectual disability, who might suffer from these psychiatric illnesses at a higher rate than the general population. Thus, one possible explanation is that, owing to intellectual and communication limitations, it is difficult to use the psychiatric diagnostic interview and apply standard diagnostic criteria to this population (Sovner and Hurley, 1983). On the other hand, the differences in species characteristics might be the reason for the different phenotypes among seipin-KO mice, seipin-knockout rats and humans with CGL2. For example, individuals with CGL2 exhibit severe lipoatrophy (a near absence of adipose tissue) (Agarwal and Garg, 2003) and seipin-knockout rats lost more than 95% of the fat mass in both subcutaneous and intra-abdominal areas (Ebihara et al., 2015), whereas more than 20% of the fat mass in WT control mice was preserved in seipin-sKO mice (Chen et al., 2012; Cui et al., 2011). In addition, the expression of low-density lipoprotein receptor is increased in the liver of seipin-deficient mice (Prieur et al., 2013) but not in seipin-knockout rats (Ebihara et al., 2015). Therefore, further experiments will be required to examine whether the impairment of hippocampal neurogenesis in seipin-nKO mice affects spatial cognitive function, which can help in understanding the mechanisms underlying the intellectual deficiency in individuals with CGL2.

## MATERIALS AND METHODS

### Animals

All animal handling procedures followed the guidelines for Laboratory Animal Research of Nanjing Medical University. The use of animals was approved by the Institutional Animal Care and Use Committee of Nanjing Medical University. The mice were maintained in a constant environmental condition (temperature 23±2°C, humidity 55±5% and 12:12 h light/dark cycle). They received a standard laboratory diet before and after all procedures. The seipin-nKO mice were generated as described elsewhere (Cui et al., 2011). Twelve-week-old WT mice (male 176 and female 40) and seipin-nKO mice (male 204 and female 40) were used at the beginning of all experiments.

### Behavioral examination

#### Forced swim test

Each mouse was placed in a glass cylinder (300 mm high, 280 mm in diameter) filled with water to a height of 20 cm (25±1°C). Mice were forced to swim for 15 min and subjected to a 6 min swimming test 24 h later. Total immobility time (minimal movements to keep the head above water) was calculated (Zhou et al., 2014).

#### Tail suspension test

Mice were suspended by the tail using adhesive tape to a rod 60 cm above the floor as described previously (Steru et al., 1985). The trials were conducted for 6 min, during which the duration of immobility was recorded.

### Histological examination and quantitative analyses

#### BrdU immunostaining

BrdU (Sigma-Aldrich, St Louis, MO, USA) was dissolved freshly in 0.9% saline to make 10 mg/ml solution just before injection. Mice were given three independent injections [intraperitoneally (i.p.)] of BrdU (50 mg/kg body weight) with an interval of 8 h between each. The mice were anesthetized with chloral hydrate (400 mg/kg body weight, i.p.) and perfused transcardially with 4% paraformaldehyde. Brains were coronally cut (40 μm) using a vibrating microtome (Microslicer DTK 1500; Dousaka EM Co., Japan). The free-floating sections were treated with 2 M HCl for 30 min at 37°C. The sections were treated with 3% normal goat serum, and then incubated in mouse monoclonal anti-BrdU antibody (1:1000; Millipore, Billerica, MA, USA) at 4°C overnight.

#### Doublecortin (DCX) and seipin immunostaining

The sections were incubated in goat polyclonal anti-DCX antibody (1:500; doublecortin C-18, Santa Cruz, CA, USA) and rabbit polyclonal anti-seipin antibody (1:1000) (Jiang et al., 2014) at 4°C overnight. After PBS rinses, the sections were incubated in biotin-labeled secondary antibody (1:500; Bioworld Technology, Inc., St Louis Park, MN, USA) for 2 h. Immunoreactivities were visualized by avidin-biotin horseradish peroxidase complex (ABC Elite; Vector Laboratories, Inc., Burlingame, CA, USA) using 3,3′-diaminobenzidine as chromogen. The immune-positive cells/fibers were observed using a conventional light microscope (Olympus DP70, ×60).

#### Nestin/GFAP immunostaining

The free-floating sections were incubated in rabbit monoclonal anti-nestin antibody (1:500; Millipore), which was revealed using CY3-labeled anti-rabbit IgG antibody (1:200) and mouse monoclonal anti-glial fibrillary acidic protein (GFAP) antibody (1:200; Millipore), which was revealed using FITC-labeled anti-mouse antibody (1:50).

#### BrdU/NeuN or GFAP immunostaining

The sections were incubated with rat monoclonal anti-BrdU antibody (1:200; Abcam, Cambridge, UK), which was revealed using CY3-labeled anti-rat IgG antibody (1:200) and mouse monoclonal anti-neuronal nuclei (NeuN) antibody (1:500, Millipore), which was revealed using fluorescein-labeled anti-mouse antibody (1:50) or mouse monoclonal anti-GFAP antibody (1:200, Millipore), which was revealed using FITC-labeled anti-mouse antibody (1:50). The immunopositive cells/fibers were observed by a confocal laser-scanning microscope (Leica, Heidelberg, Germany).

#### Quantitative evaluation of immunopositive cells/fibers

(1) The number of BrdU^+^ cells in every 5th section (200 μm apart) was obtained from 12 sections and multiplied by 5 to give the total number in each DG. (2) Nestin^+^ fibers (red) and both Nestin^+^/GFAP^+^ fibers (yellow) in the granular cell layer (GCL) were analyzed with the software ImageJ (NIH Image, Bethesda, MD, USA) to obtain the density of Nestin^+^/GFAP^+^ and Nestin^+^/GFAP^−^ fibers (number per mm^3^), respectively (Luo et al., 2010). (3) DCX^+^ cells in the subgranular zone (SGZ) and DCX^+^ fibers in the middle molecular layer (mML) were counted to obtain the density of DCX^+^ cells (number of DCX^+^ cells per mm) and DCX^+^ fibers (number of DCX^+^ fibers per DCX^+^ cell), respectively (Sha et al., 2014).

### Drug administration

The PPARγ agonist rosiglitazone (rosi; Enzo, Farmingdale, NY, USA) was dissolved in dimethyl sulfoxide (DMSO), and then diluted in 0.9% saline to a final concentration of 0.5% DMSO. Oral administration (p.o.) of rosi (5 mg/kg body weight) was given daily (Salehi-Sadaghiani et al., 2012). The MEK inhibitor U0126 (Sigma-Aldrich, USA) dissolved in 0.5% DMSO was injected (i.c.v., 0.3 nmol/mouse/day) (Yang et al., 2012). For repeated i.c.v. injection, a 26-G stainless-steel guide cannula (Plastics One, Roanoke, VA, USA) was implanted into the right lateral ventricle (0.3 mm posterior to bregma, 1.0 mm lateral, and 2.3 mm ventral) and anchored to the skull with three stainless steel screws and dental cement.

### Reverse transcription-polymerase chain reaction (RT-PCR)

Total RNA was isolated from the hippocampus and cortex with TRIzol reagent (Invitrogen, Camarillo, CA, USA) and reverse-transcribed into cDNA using a Prime Script RT reagent kit (Takara, China) for quantitative PCR (ABI Step One Plus, Foster City, CA, USA) in the presence of a fluorescent dye (SYBR Green I; Takara). The relative expression of genes was determined using the 2−ΔΔct method with normalization to GAPDH expression. Primer sets were used as listed in the Table S1.

### Western blot analysis

Mice were decapitated under anesthesia with chloral hydrate. Hippocampus was taken quickly and homogenized in a lysis buffer containing 50 mM Tris-HCl (pH 7.5), 150 mM NaCl, 5 mM EDTA, 10 mM NaF, 1 mM sodium orthovanadate, 1% Triton X-100, 0.5% sodium deoxycholate, 1 mM phenylmethylsulfonyl fluoride and protease inhibitor cocktail. Total proteins (20 μg) were separated by SDS-polyacrylamide gel electrophoresis and transferred to a polyphorylated difluoride membrane. The membranes were incubated with rabbit monoclonal antibodies of phosphorylated ERK1/2 and STAT3 (1:1000; Cell Signaling Technology, Inc., Boston, MA, USA) or Wnt3 (1:2000; Millipore) at 4°C overnight. The membranes were incubated with HRP-labeled secondary antibodies. The blots were stripped by incubation in stripping buffer (Restore, Pierce) for 5 min, incubated with rabbit monoclonal antibodies of ERK1/2 or STAT3 (1:1000). Western blot bands were scanned and analyzed with the image analysis software package (ImageJ; NIH Image, Bethesda, MD, USA).

### Analysis of lipids

Lipids were extracted from the hippocampus as described previously (Le Belle et al., 2001). The hippocampus was quickly freeze-clamped in liquid nitrogen, and then pulverized by a stainless steel mortar and pestle. After the addition of chloroform:methanol (1:2), the sample was mixed for 1 min, and then decanted in a vacuum container with rotary shaking at 4°C for 2 h. The sample added with chloroform and water was incubated on ice for 1 min. After centrifugation at 12,100 ***g*** for 3 min at 4°C, the lower organic phase was collected. Subsequently, 1 M HCl and chloroform were added to the remainder, and incubated on ice for 3 min. The lower organic phase collected after centrifugation at 12,100 ***g*** for 3 min at 4°C was combined with the first extract. The extracted lipids were blown dry with nitrogen gas, and again suspended in solvent for mass spectrometry analysis. Lipidomic analysis and quantitative measurement of neutral lipids were performed by high-performance liquid chromatography electrospray tandem mass spectrometry (HPLC-ES/MS), according to the method described by Jiang et al. (2014).

### Statistical analysis

The group data were expressed as the mean±s.e.m. All statistical analyses were performed using SPSS software, version 16.0 (SPSS Inc., USA). Differences among means were analyzed using the Student's *t*-tests or analyses of variance (ANOVA) with or without repeated measures, followed by Bonferroni post-hoc analysis. Differences at *P*<0.05 were considered statistically significant.
